# Supplementary material for: Neutrophil extracellular trap formation requires OPA1-dependent glycolytic ATP production
Source: Nat Commun. 2018 Jul 27;9:2958. doi: 10.1038/s41467-018-05387-y (PMC6063938; doi:10.1038/s41467-018-05387-y)
Supplement: Supplementary file 1 — Supplementary Information [file 41467_2018_5387_MOESM1_ESM.pdf]

Supplementary Figures for “Neutrophil extracellular trap formation requires OPA1-dependent glycolytic ATP production” by Amini and Stojkov *et al*.

Supplementary Figures 1 - 11

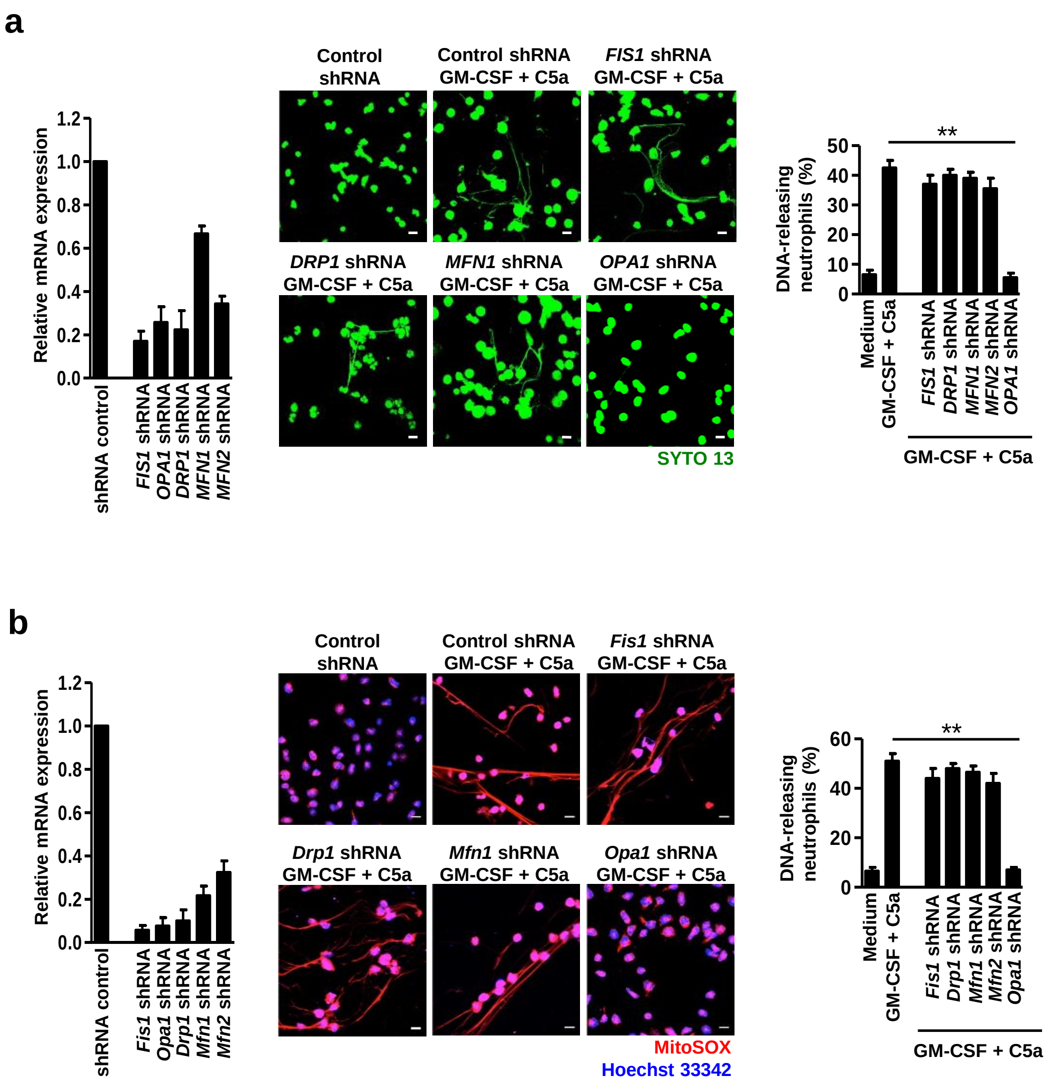

Supplementary Figure 1

Down-regulation of OPA1 expression prevents DNA release from activated differentiated PLB-985 and Hoxb8 neutrophils. (**a**) PLB-985 cells. *Left:* Quantitative real-time PCR. Following transduction with lentiviruses encoding control or *FIS1*, *DRP1*, *MFN1*, and *OPA1* shRNA, single cell clones were isolated after puromycin selection and subsequently analyzed. S18 was used as a reference gene to normalize the expression of the specific genes. The percentage of mRNA reduction was calculated and compared to control shRNA-treated cells. *Middle:* Following differentiation, the genetically modified PLB-985 cells were primed with GM-CSF and subsequently stimulated with C5a. DNA was stained with Syto 13 (green). Bars, 10 µm. *Right:* Quantification of the DNA-releasing cells. Values are means ± SEM. **, p<0.01; n=3. (**b**) Hoxb8 mouse neutrophils. *Left:* Quantitative real-time PCR. Undifferentiated Hoxb8 neutrophils were transduced with virus encoding *Fis1*, *Drp1*, *Mfn1*, *Opa1* and control shRNA. Single cell clones were isolated after G418 selection and subsequently analyzed. S18 was used as a reference gene to normalize the expression of the specific genes. The percentage of mRNA reduction was calculated and compared to control shRNA-treated cells. *Middle:* Following differentiation, the genetically modified mature Hoxb8 neutrophils were primed with GM-CSF and subsequently stimulated with C5a. The extracellular DNA was stained with MitoSOX™ Red and the nucleus with Hoechst 33342 (blue). Bars, 10 µm. *Right:* Quantification of the DNA-releasing neutrophils. Values are means ± SEM. **, p<0.01; n=3.

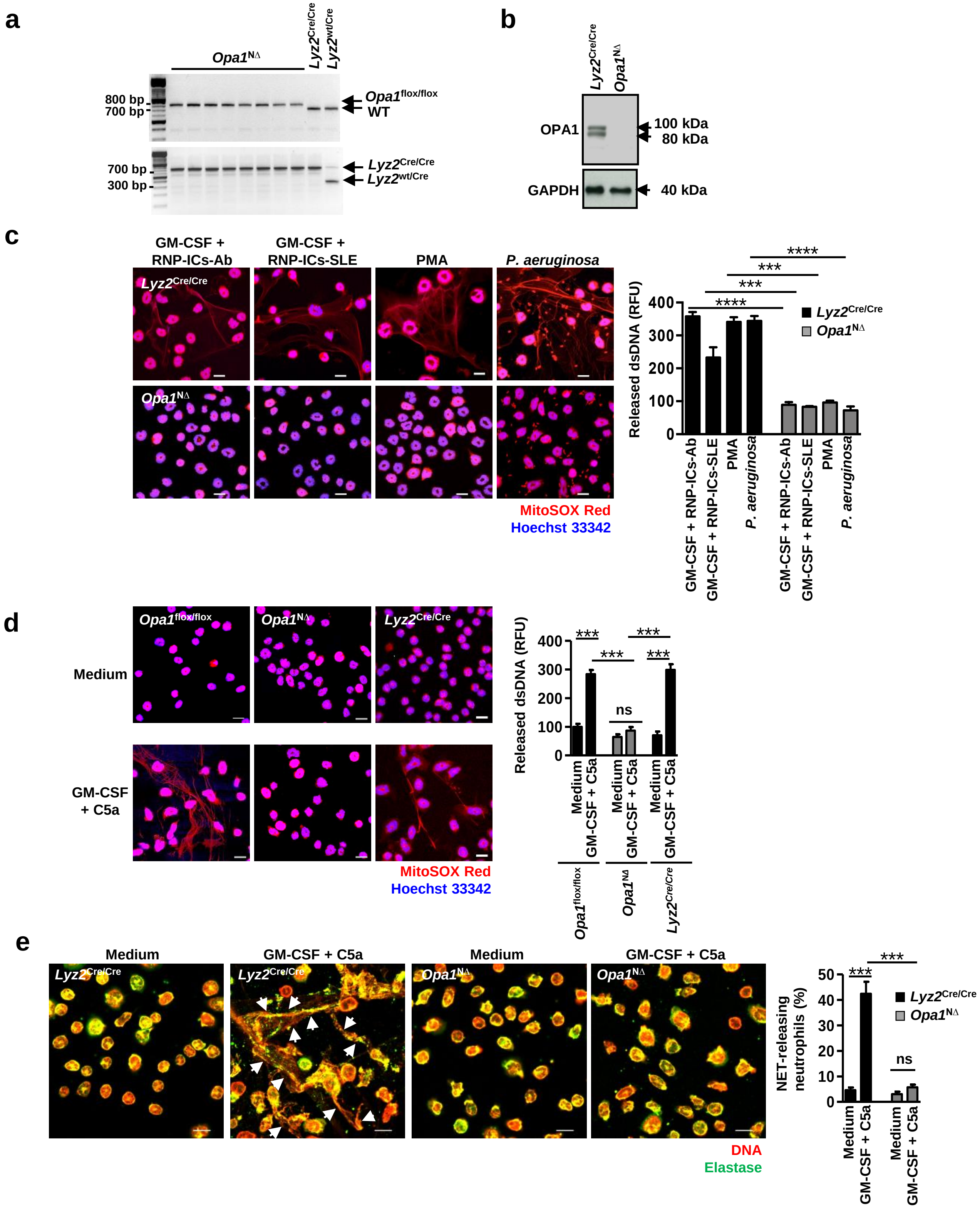

Supplementary Figure 2

Opa1-knockout (*Opa1*NΔ) primary mouse neutrophils are unable to release DNA following activation.(**a**) Genotyping of *Opa1*NΔ mice. Genomic DNA was extracted from ear biopsies and tested by PCR. (**b**) Immunoblotting. Freshly purified mouse neutrophils of *Opa1*NΔ and control mice were analyzed for OPA1 protein expression. (**c**) Confocal microscopy. Primary mature neutrophils from *Opa1*NΔ and control mice were primed with GM-CSF and subsequently stimulated with two different immune complexes (RNP-ICs-Ab or RNP ICs-SLE). Alternatively, neutrophils were activated with PMA or *P. aeruginosa* in the absence of priming. Extracellular DNA was stained with MitoSOX™ Red and the nucleus with Hoechst 33342 (blue). Bars, 10 μm. *Right:* Quantification of released dsDNA in supernatants of activated neutrophils. Values are means ± SEM. ***, p<0.001; ****, p<0.0001; n=5. (**d**) Confocal microscopy. Primary mature neutrophils from *Opa1*NΔ and control mice (*Opa1*flox/flox and *Lyz2*Cre/Cre mice) were primed with GM-CSF and subsequently stimulated with C5a. Extracellular DNA was stained with MitoSOX™ Red and the nucleus with Hoechst 33342 (blue). Bars, 10 μm. *Right:* Quantification of released dsDNA in supernatants of activated neutrophils. Values are means ± SEM. ***, p<0.001; n=3. (**e**) Confocal microscopy. Primary mature mouse neutrophils from *Opa1*NΔ and control mice were primed with GM-CSF and subsequently stimulated with C5a. NET formation was assessed by demonstrating co-localization of elastase (green) and DNA (red) (white arrows) using anti-elastase and anti-dsDNA antibodies. Bars, 10 μm. *Right:* Quantification of NET-forming neutrophils. Values are means ± SEM. ***, p<0.001; n=4.

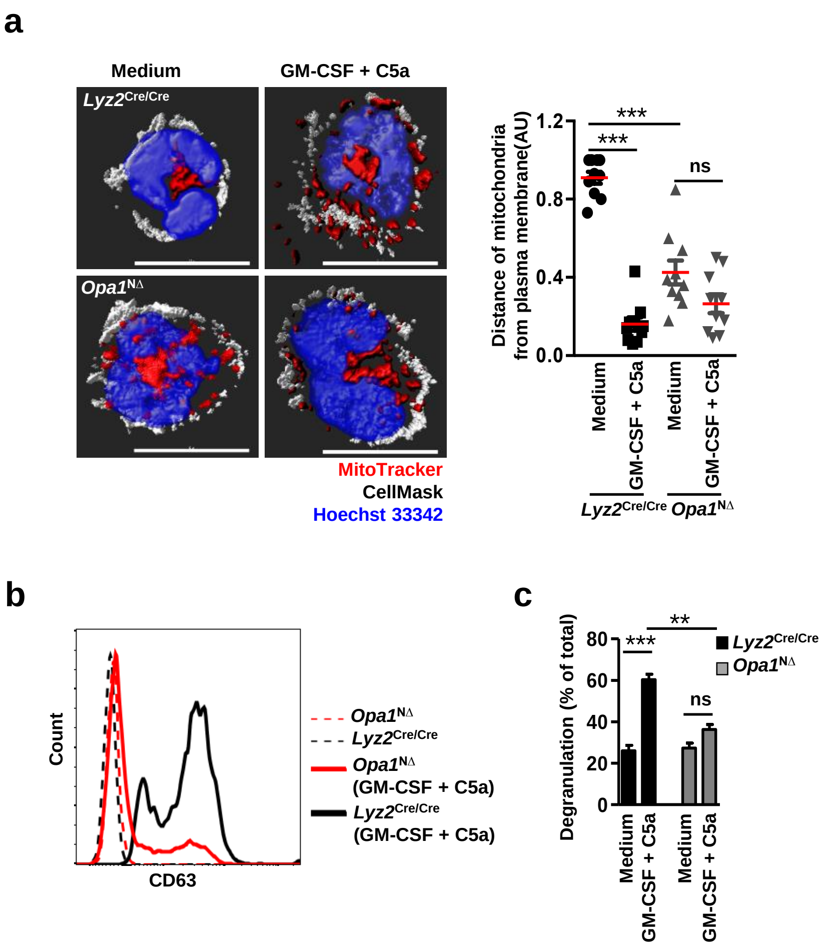

Supplementary Figure 3

Opa1-knockout (*Opa1*$^{N\Delta}$) primary mouse neutrophils are unable to translocate mitochondria nor to exhibit detectable degranulation after activation.. (**a**) Confocal microscopy. *Opa1*$^{N\Delta}$ and *Lyz2*$^{Cre/Cre}$ control neutrophils were primed with GM-CSF and subsequently stimulated with C5a. Mitochondria were stained with MitoTracker® Orange (red), cell membrane with CellMask (white), and the nucleus with Hoechst 33342 (blue). Iso-surface reconstruction was performed using the Imaris software. Bars, 10 µm. *Right:* Statistical analysis was performed by analyzing ten representative pictures from each condition, each containing more than 20 cells. Values are means ± SEM. **, p<0.01; ***, p<0.001; n=3. (**b**) Flow cytometry. Degranulation of azurophilic granules was determined using the surrogate marker CD63. *Opa1*$^{N\Delta}$ and *Lyz2*$^{Cre/Cre}$ control neutrophils were primed with GM-CSF and subsequently stimulated with C5a. Representative data of three independent experiments are shown. (**c**) Degranulation of mouse neutrophils was assessed by measuring the activity of β-glucosaminidase released from neutrophils. Neutrophils were primed with GM-CSF and subsequently stimulated with C5a. Values are means ± SEM. **, p<0.01; ***, p<0.001; n=3.

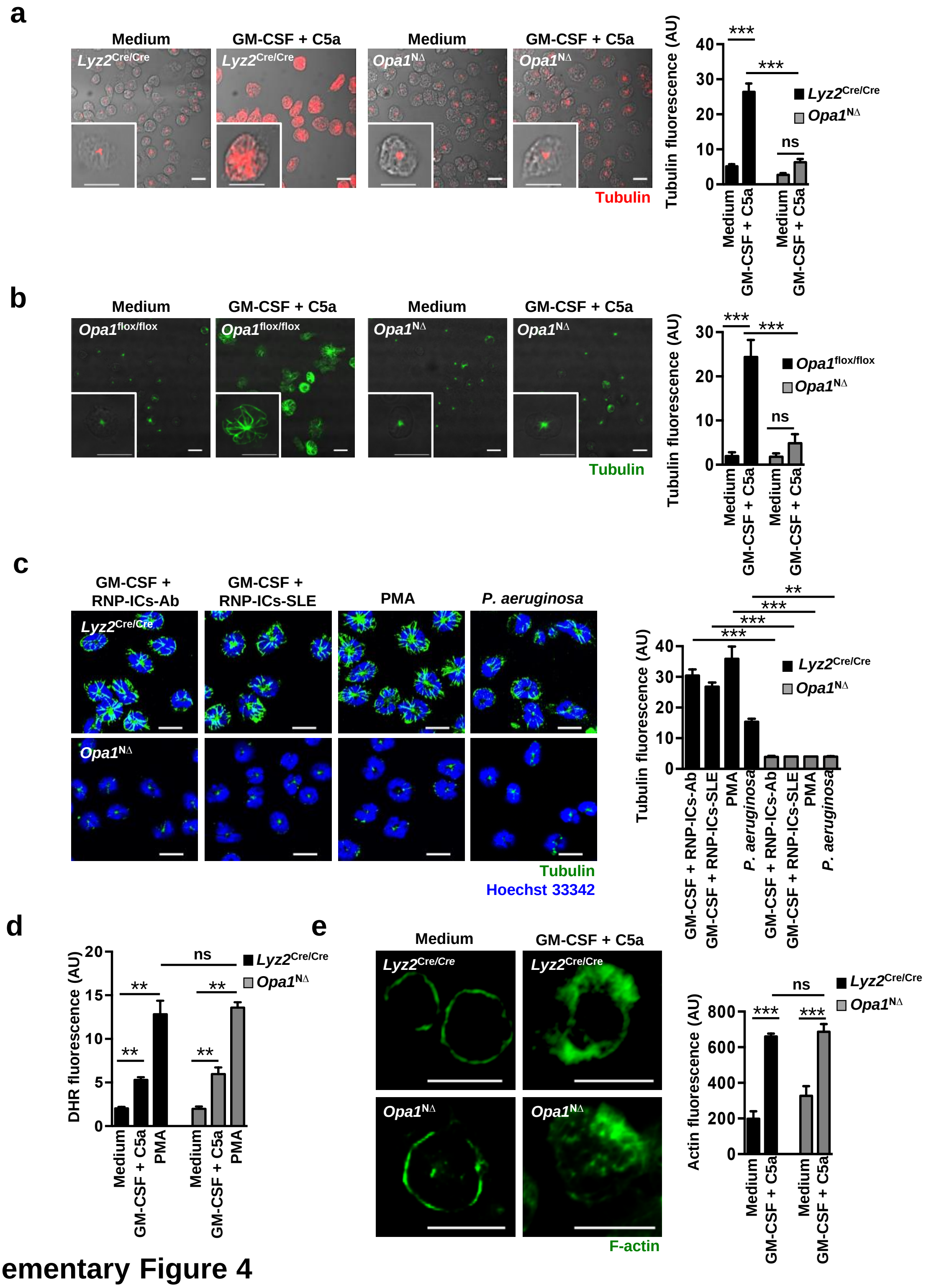

Supplementary Figure 4

Opa1-knockout (*Opa1*$^{N\Delta}$) primary mouse neutrophils are unable to form a microtubule network after activation, but exhibit no defect in actin polymerization. (**a**) Confocal microscopy. *Opa1*$^{N\Delta}$ and *Lyz2*$^{Cre/Cre}$ control neutrophils were primed with GM-CSF and subsequently stimulated with C5a. Microtubules were stained with anti-α-tubulin antibody (red). Bars, 10 µm. *Right:* Quantification of microtubule network formation was performed by automated analysis of microscopic images using Imaris software. Values are means ± SEM. ***, p<0.001; n=5. (**b**) Confocal microscopy. *Opa1*$^{N\Delta}$ and *Opa1*$^{flox/flox}$ control neutrophils were primed with GM-CSF and subsequently stimulated with C5a. Microtubules were stained with anti-α-tubulin antibody (green). Bars, 10 µm. *Right:* Quantification of microtubule network formation was performed by automated analysis of microscopic images using Imaris software. Values are means ± SEM. ***, p<0.001; n=3. (**c**) Confocal microscopy. GM-CSF primed neutrophils of *Opa1*$^{N\Delta}$ and *Lyz2*$^{Cre/Cre}$ control neutrophils were stimulated with two different immune complexes (RNP-ICs-Ab or RNP ICs-SLE). Alternatively, neutrophils were stimulated with PMA or *P. aeruginosa* in the absence of priming. Microtubules were stained with anti-α-tubulin antibody (green) and the nucleus with Hoechst 33342 (blue). Bars, 10 µm. *Right:* Quantification of microtubule network formation was performed by automated analysis of microscopic images using Imaris software. Values are means ± SEM. **, p<0.01; ***, p<0.001; n=4. (**d**) Flow cytometry. The detection of ROS production was based on the oxidation of DHR 123. *Opa1*$^{N\Delta}$ and *Lyz2*$^{Cre/Cre}$ control neutrophils were primed with GM-CSF and subsequently stimulated with C5a. PMA (25 nM) stimulation served as a positive control. Values are means ± SEM. **, p<0.01; n=3. (**e**) Confocal microscopy. F-actin formation was analyzed in *Opa1*$^{N\Delta}$ and *Lyz2*$^{Cre/Cre}$ control neutrophils following GM-CSF priming and subsequent C5a stimulation. Bars, 10 µm. *Right:* Quantification of F-actin formation was performed by automated analysis of microscopic images using Imaris software. Values are means ± SEM. ***, p<0.001; n=5.

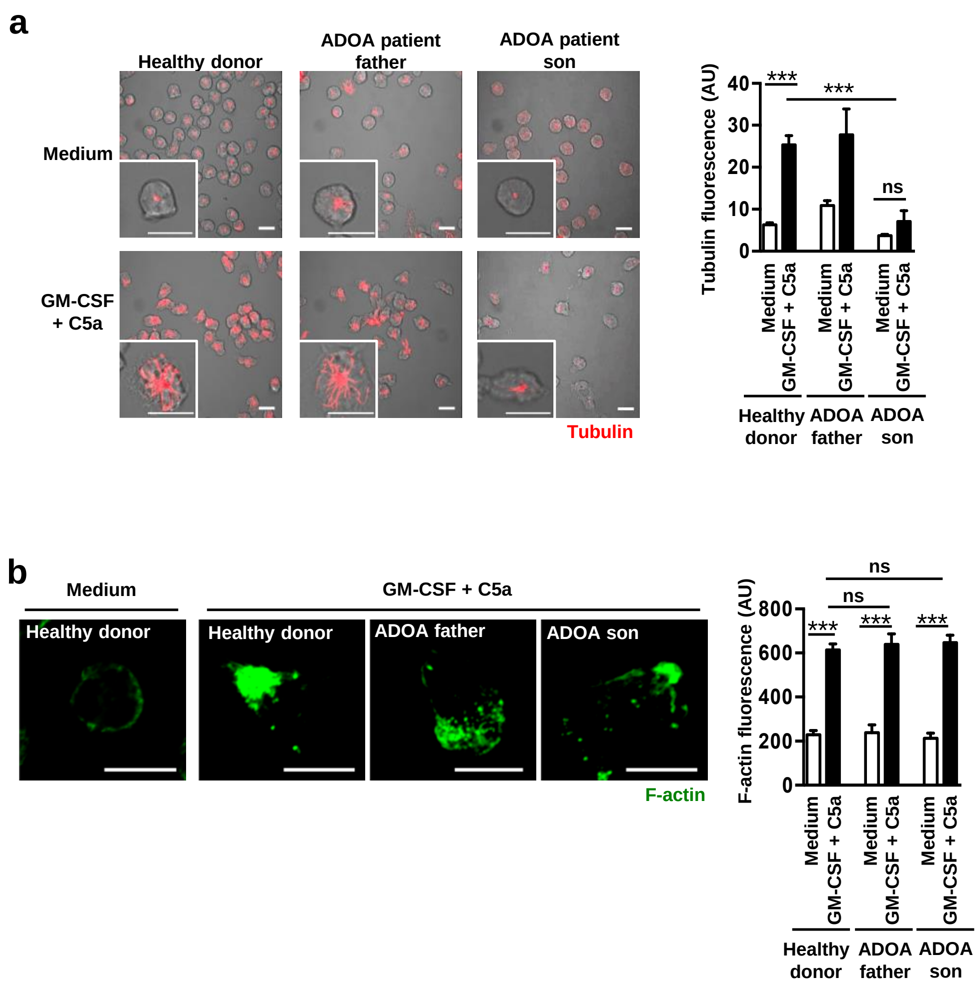

Supplementary Figure 5

OPA1-deficient human neutrophils are unable to form a microtubule network after activation, but exhibit no defect in actin polymerization. (**a**) Confocal microscopy. Microtubule formation of human blood neutrophils from control individuals and two ADOA patients following GM-CSF priming and subsequent C5a stimulation. Microtubules were stained with anti-α-tubulin antibody (red). Bars, 10 µm. *Right:* Quantification of microtubule network formation was performed by automated analysis of microscopic images using Imaris software. Values are means ± SEM. ***, $p<0.001$; n=3. (**b**) Confocal microscopy. F-actin formation was analyzed in human blood neutrophils from control individuals and two ADOA patients following GM-CSF priming and subsequent C5a stimulation. Bars, 10 µm. *Right:* Quantification of F-actin formation was performed by automated analysis of microscopic images using Imaris software. Values are means ± SEM. ***, $p<0.001$; n=3.

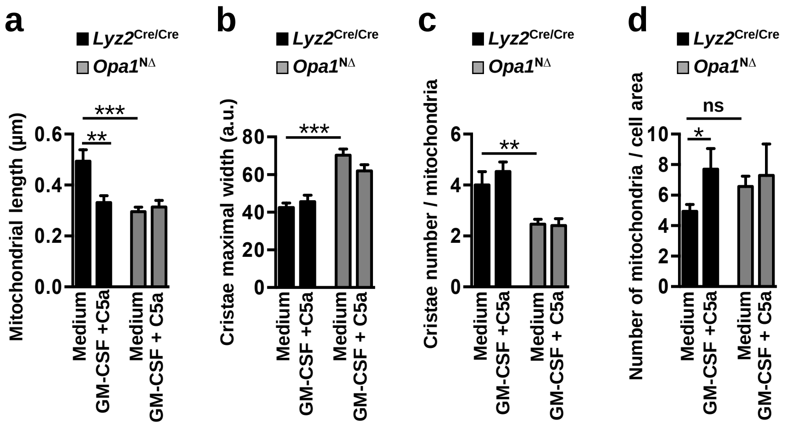

Supplementary Figure 6

Lack of *Opa1* prevents an increase in mitochondrial numbers and reduction in mitochondrial length upon neutrophil activation. (**a**) Transmission electron microscopy (TEM). Average mitochondrial major axis length in freshly purified mature neutrophils from *Opa1*$^{N\Delta}$ and control mice in the presence and absence of GM-CSF priming and subsequent C5a stimulation. Images were acquired by TEM and subsequently analyzed using the measurement points module of Imaris software. Data were collected from at least five mitochondria per cell from more than 50 neutrophils per experiment. Values are means ± SEM. ***, $p<0.001$; n=3. (**b**) Morphometric analysis of cristae width in 60 randomly selected mitochondria of freshly purified mature neutrophils from *Opa1*$^{N\Delta}$ and control mice in the presence and absence of GM-CSF priming and subsequent C5a stimulation. Images were acquired by TEM and subsequently analyzed using the measurement points module of Imaris software. Values are means ± SEM. ***, $p<0.01$; n=3. (**c**) The average number of cristae per mitochondria were counted in 60 randomly selected mitochondria of freshly purified mature neutrophils from *Opa1*$^{N\Delta}$ and control mice in the presence and absence of GM-CSF priming and subsequent C5a stimulation. Images were acquired by TEM. Values are means ± SEM. ***, $p<0.01$; n=3. (**d**) Freshly purified mature neutrophils from *Opa1*$^{N\Delta}$ and control mice were analyzed in the presence and absence of GM-CSF priming and subsequent C5a stimulation. Images were acquired by TEM and the average number of mitochondria per neutrophil was quantified in at least 100 cells. Values are means ± SEM. *, $p<0.05$; n=3.

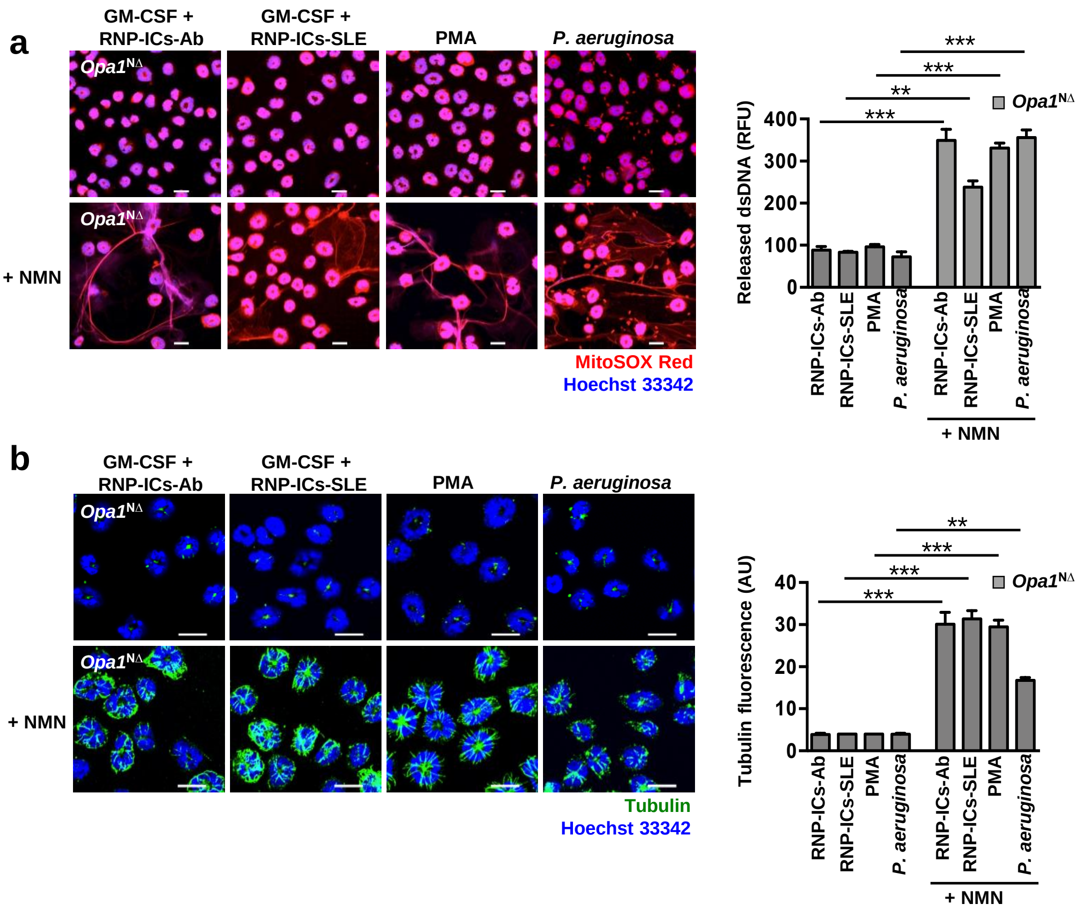

Supplementary Figure 7

NMN restores the ability of *Opa1*-knockout (*Opa1*$^{N\Delta}$) primary mouse neutrophils to release DNA and to form the microtubule network after activation with various stimuli. (**a**) Confocal microscopy. Primary mature neutrophils from *Opa1*$^{N\Delta}$ and control mice were primed with GM-CSF and subsequently stimulated with two different immune complexes (RNP-ICs-Ab or RNP ICs-SLE). Alternatively, neutrophils were stimulated with PMA or *P. aeruginosa* in the absence of priming. In the lower panel, neutrophils were pretreated with 500 µM NMN. Extracellular DNA was stained with MitoSOX™ (red) and the nucleus with Hoechst 33342 (blue). Bars, 10 µm. *Right:* Quantification of released dsDNA in supernatants of activated neutrophils. Values are means ± SEM. **, p<0.01; ***, p<0.001; n=5. (**b**) Confocal microscopy. Primary mature neutrophils from *Opa1*$^{N\Delta}$ and control mice were primed as described in (a). In the lower panel, neutrophils were pretreated with 500 µM NMN. Microtubules were stained with anti-α-tubulin antibody (green) and the nucleus with Hoechst 33342 (blue). Bars, 10 µm. *Right:* Quantification of the microtubule network formation was performed by automated analysis of microscopic images using Imaris software. Values are means ± SEM. **, p<0.01; ***, p<0.001; n=3.

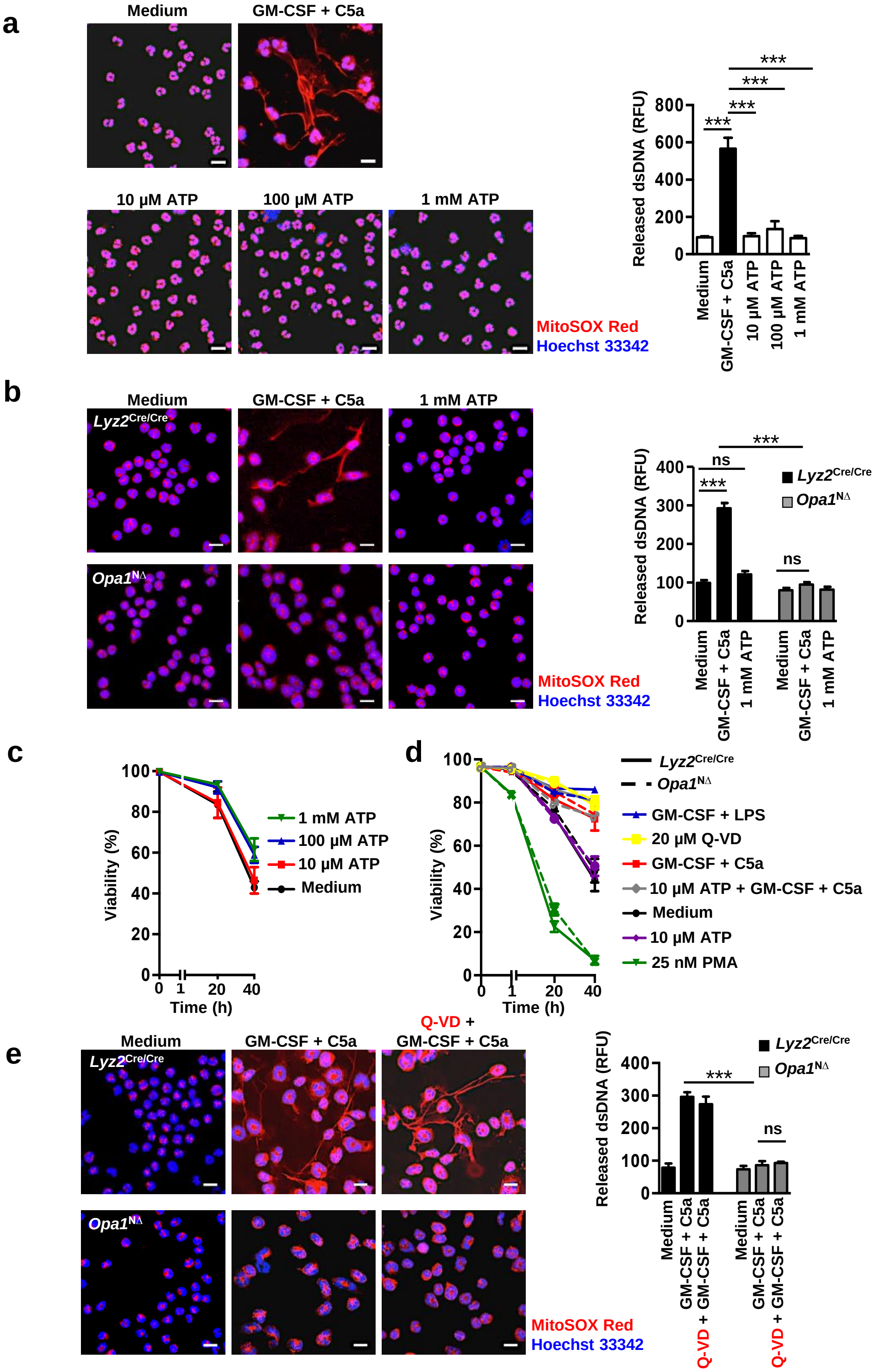

Supplementary Figure 8

ATP does not directly trigger extracellular DNA release in human and mouse neutrophils. (**a**) Human blood neutrophils were incubated with the indicated concentrations of ATP. As a positive control, neutrophils were primed with GM-CSF and subsequently stimulated with C5a. Extracellular DNA was stained with MitoSOX™ Red and the nucleus with Hoechst 33342 (blue). Bars, 10 µm. *Right:* Quantification of released dsDNA in supernatants of activated neutrophils. Values are means ± SEM. ***, p<0.001; n=3. (**b**) Confocal microscopy. Primary mature neutrophils from *Opa1*$^{N\Delta}$ and control mice were incubated with 1 mM ATP. As a positive control, neutrophils were primed with GM-CSF and subsequently stimulated with C5a. Extracellular DNA was stained with MitoSOX™ Red and the nucleus with Hoechst 33342 (blue). Bars, 10 µm. *Right:* Quantification of released dsDNA in supernatants of activated neutrophils. Values are means ± SEM. ***, p<0.001; n=3. (**c**) Flow cytometry. Human blood neutrophils were cultured under the indicated conditions and viability assessed after the indicated times. Values are means ± SEM (n=3). (**d**) Flow cytometry. Primary mature neutrophils from *Opa1*$^{N\Delta}$ and control mice were cultured under the indicated conditions and viability assessed after the indicated times. Values are means ± SEM (n=3). **(e)** Confocal microscopy. Primary mature neutrophils from *Opa1*$^{N\Delta}$ and control mice were primed with GM-CSF and subsequently stimulated with C5a in the presence of 20 µM Q-VD, a pan-caspase inhibitor. Extracellular DNA was stained with MitoSOX™ Red and the nucleus with Hoechst 33342 (blue). Bars, 10 µm. *Right:* Quantification of released dsDNA in supernatants of activated neutrophils. Values are means ± SEM. ***, p<0.001; n=3.

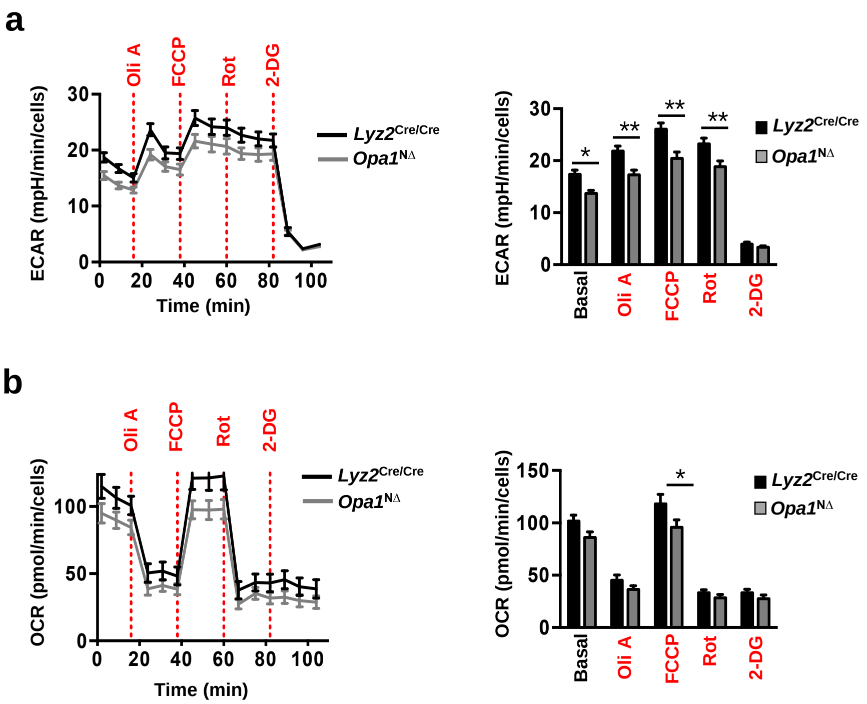

Supplementary Figure 9

The effect of OPA1 depletion on extracellular acidification rate (ECAR) and oxygen consumption rate (OCR) in primary mouse neutrophils. During extracellular flux analysis, neutrophils were sequentially treated with oligomycin A (Oli A), carbonyl cyanide-4-(trifluoromethoxy) phenylhydrazone (FCCP), rotenone (Rot) and 2-deoxy-d-glucose (2-DG) to assess different glycolytic parameters (**a**) or to detect different OXPHOS parameters (**b**). Values are means ± SEM. *, $p<0.05$ **; $p<0.01$; n=3.

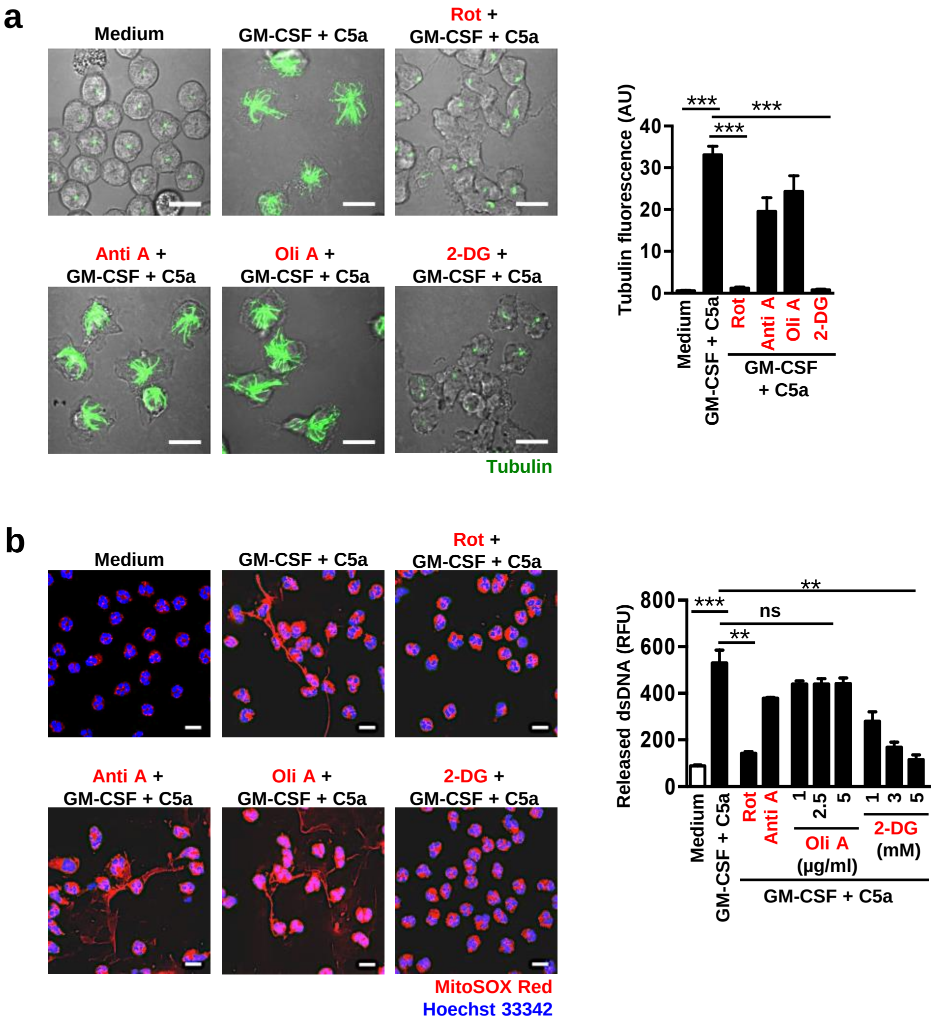

Supplementary Figure 10

Pharmacological inhibition of mitochondrial complex I activity and glycolysis blocks microtubule network formation and extracellular DNA release in human neutrophils. (**a**) Confocal microscopy. Human blood neutrophils were pretreated with 10 µM rotenone, 5 µg per ml antimycin A, 2.5 µg per ml oligomycin A, or 3 mM 2-DG for 30 min before GM-CSF priming and subsequent C5a activation. Microtubules were stained with anti-α-tubulin antibody (green). Bars, 10 µm. *Right:* Quantification of microtubule network formation was performed by automated analysis of microscopic images using Imaris software. Values are means ± SEM. ***, $p<0.001$; n=3. (**b**) Confocal microscopy. Human blood neutrophils were pretreated with 10 µM rotenone, 5 µg per ml antimycin A, 2.5 µg per ml oligomycin A, or 3 mM 2-DG for 30 min before GM-CSF priming and subsequent C5a activation. Extracellular DNA was stained with MitoSOX™ Red and the nucleus with Hoechst 33342 (blue). Bars, 10 µm. *Right:* Quantification of released dsDNA in supernatants of activated neutrophils. Values are means ± SEM. **, $p<0.01$; ***, $p<0.001$; n=3.

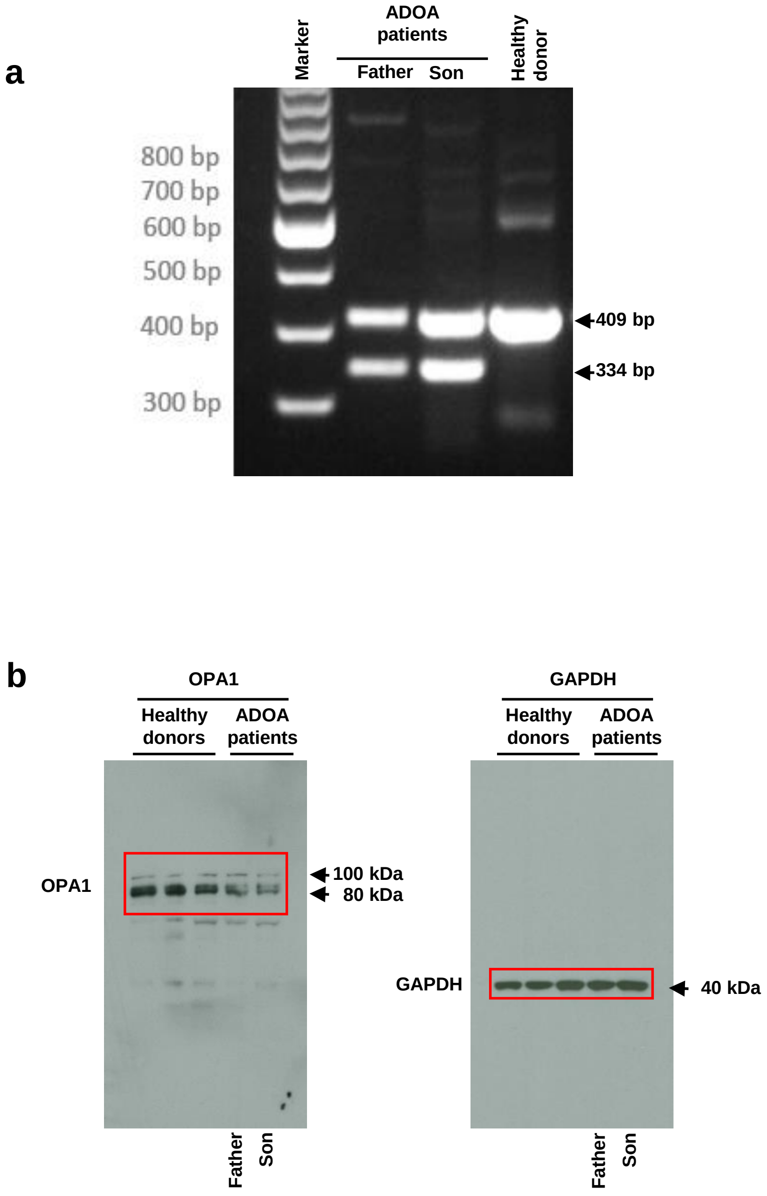

Supplementary Figure 11

Full images of gel electrophoresis and immunoblots. (**a**) Un-cropped gel image of Fig. 1a. Molecular characterization of *OPA1* transcripts in ADOA patients. *OPA1* transcript analysis from isolated neutrophils encompassing exons 10-12 derived from a control and two ADOA patients harboring the heterozygous c.1140G>A mutation (NM_015560.2). A shortened product of 334 bp reveals the aberrantly spliced, skipped exon 11 and is detected in patients' cDNA only. The band of 409 bp indicates the wild-type transcript. (**b**) Un-cropped immunoblot image of Fig. 1c. Immunoblotting. Protein lysates of freshly isolated neutrophils from ADOA patients and healthy donors were analyzed for OPA1 protein expression; *left panel*, and GAPDH; *right panel*.
